# Relationships between the Physical Activity Intensity and the Medical Expenditure of Middle-Aged and Elderly People: Parsing from the CHARLS Database

**DOI:** 10.3390/bs13070566

**Published:** 2023-07-07

**Authors:** Linhong Chen, Xiaocang Xu

**Affiliations:** 1 School of Marxism, Chongqing Technology and Business University, Chongqing 400067, China; chenlinhong@ctbu.edu.cn; 2 School of Economics and Management, Huzhou University, Huzhou 313000, China

**Keywords:** medical expenditure, physical activity (PA), middle-aged and elderly people, two-part model

## Abstract

There are many studies on the impact of physical activity on health but few studies on the relationship between physical activity and medical expenditure among the elderly. Based on the China Health and Retirement Longitudinal Survey (CHARLS) database and selected 4456 valid samples, this paper used a two-part model to analyze the effects of high, moderate, and low physical activity intensity on medical expenditure. It is found that the intensity of physical activity was negatively correlated with medical expenditure, and the medical expenditure of the high physical activity intensity group was significantly lower than that of the low physical activity intensity group. For example, compared to people with no physical activity, the total medical expenditure decreased by 22.4%, 40.4%, and 62.5% per week in those with low, moderate, and high physical activity intensity. Thus, the government should provide more places for the elderly to exercise, planning special exercise areas for the elderly in community playgrounds, such as a dancing square, which will also help the elderly to increase their amount of exercise per week and develop a daily exercise habit.

## 1. Introduction

The aging of the population is a far-reaching global problem that poses a severe challenge not only to the health of the elderly but also to the government’s medical expenditure policy. The U.S. spends about USD 3.6 trillion on healthcare, accounting for 18% of the GDP (USD 4030 per capita), while China spends only 6% (USD 214 per capita), far less than the growing demand for healthcare (National Bureau of Statistics of China, 2021). In China, the number of medical visits was 1.286 billion in 2000 and 3.884 billion in 2021. The overall growth rate from 2000 to 2021 is 202.02%, with an average annual growth rate of 118 million people. At the same time, China’s per capita medical expenditure is on the rise, from 2000 yuan in 2015 to more than 3500 yuan in 2019 (China Health Statistical Yearbook, 2021). Medical expenditure needs are closely related to the physical health status of the elderly, and physical activity (PA) directly affects health status [1]. Therefore, physical activity (PA) and medical expenditure also form a certain correlation.

Scholarly research on medical expenditure flourished in the 1990s [2], mainly focusing on the measurement and influencing factors of medical expenditure within the background of population aging, such as income level, education level, medical security system, etc. First of all, the level of household income and medical expenditure change in the same direction, which was proven by Newhouse (1992) [3] and Reeves and Kee (2016) [4]. In China, in addition to discussing the impact of income on medical expenditure [5], some scholars specifically discussed the urban–rural difference in the impact of income on medical expenditure [6]. Second, education level is also an important factor affecting medical expenditure. Mwabu et al. (1993) [7] and Hjortsberg (2003) [8], respectively, took Kenya and Zambia as examples and found that medical expenditure increased with the improvement of education levels. Some studies in China have also confirmed this finding [9], but Dai and Li (2008) [10] and Zhang et al. (2015) [11] found that there is a negative relationship between the medical care consumption of rural residents and the per capita education years. Third, medical insurance can reduce the burden of medical expenditure. Hurd and McGarry (1997) [12], Kavosi et al. (2014) [13], and Sepehri (2019) [14] have all confirmed that urban residents’ participation in medical insurance can effectively avoid catastrophic family medical expenses. Further, Chinese studies have found that there are significant urban–rural and regional differences in the role of medical insurance in promoting medical expenditure [15]. To sum up, global scholars have discussed the influencing factors of medical expenditure from different dimensions.

However, there is not much research on the impact of physical activity (PA) on medical expenditure, and it was not until the past five years that it began to attract attention. The relationship between physical activity (PA) and medical expenditure stems from the impact of physical activity (PA) on health [16,17]. Among them, differences in race are involved [18,19,20,21,22]. Moreover, the COVID-19 pandemic has given researchers something to consider ([23]). Research on the impact of physical activity (PA) on medical expenditure has received much attention in recent years. Firstly, Marashi et al. (2019) [24] and Xu et al. (2018) [25]—studies in the United States, and Watanabe et al. (2018) [26] studies in Japan, show that there is a statistically significant association between physical activity and lower hospital payments. Moreover, the cost savings associated with physical activity are significantly larger for elderly and low-income people. However, Sato et al. (2020) [27] found that physical activity was not associated with Medicare costs occurring in the concurrent and subsequent year (*p* > 0.05), but the two-year lagged variable (*p* = 0.03) and the three-year lagged variable (*p* = 0.01) for physical activity prevalence were negatively associated with Medicare costs, indicating a time-lagged relationship. Secondly, when it comes to the medical expenditure for different diseases, Wu et al. (2019) [28] found that regular exercise (>30 min at least five times weekly) can reduce the medical expenditure of obese middle-aged and elderly patients with diabetes. The same is true for people at high risk of cardiovascular disease [29]. Araujo et al. (2018) [30] found a positive correlation between physical activity and medical expenses for diabetes in 316 elderly people in a Brazilian city who were followed for 18 months. Thirdly, the impact of physical activity (PA) on medical expenditure also has gender differences. Okunrintemi et al. (2019) [31] found that the proportion of women with cardiovascular disease (CVD) not meeting the recommended physical activity level is increasing, particularly among certain racial/ethnic and socioeconomic groups, and is associated with significant medical expenditures. Gomes et al. (2020) [32] examined the twelve-year trajectories of physical activity and health costs in middle-aged Australian women.

In recent years, with the increasingly serious aging of China’s population, more and more scholars have studied the impact of physical activity on medical expenditure by taking China as a case study. Wang et al. (2019) [33], Su et al. (2020) [34], and Song et al. (2020) [35], respectively, confirmed that physical activity was associated with lower medical expenditure in middle-aged and elderly patients with coronary heart disease, diabetes mellitus, and cardiovascular disease. In addition, Liang et al. (2022) [36] used the extreme gradient-boosting model and found physical exercise status to be a robust predictor of self-rated health (SRH) outcomes in older adults, based on the data from the 2018 China Family Panel Studies survey. However, compared with the research in other countries, especially in Europe and the United States, the depth of relevant research and analysis methods in China still needs to be further explored.

In summary, there are more and more studies on huge medical expenditure and its influencing factors, but the relevant research on the impact of physical activity on medical expenditure has only begun to increase in the past five years, especially after the COVID-19 outbreak. On the basis of previous literature, this paper further studies this problem. Under the theoretical framework of the Andersen model, this paper selected the newest data of the China Health and Retirement Longitudinal Survey (CHARLS) to confirm the relationship between physical activity intensity and medical expenditure in middle-aged and elderly people, which also provides some insights for government policies.

The marginal contribution of this study is that it classified low, moderate, and high physical activity intensity and used the two-part model to confirm the relationships between the different physical activity intensities and medical expenditures in middle-aged and elderly people.

## 2. Materials and Methods

### 2.1. Data Source

The China Health and Retirement Longitudinal Survey (CHARLS) is a large-scale interdisciplinary survey project conducted by the National School of Development, Peking University, and jointly executed by the China Social Science Survey Center and the Youth League Committee of Peking University. It is a major project, funded by the national natural science foundation, that aims to collect a dataset of those over 45 years of age and the elderly families in China. The questionnaire design of CHARLS is based on international experience, including the U.S. Health and Retirement Survey (HRS), the U.K. Aging Tracking Survey (ELSA), and the European Health, Ageing and Retirement Survey (SHARE). Multi-stage sampling was adopted in the project. The PPS (probability proportion to size) sampling method was adopted in both county/district and village sampling stages. The CHARLS questionnaire included basic personal information, family structure and financial support, health status, physical measurements, medical service utilization and health insurance, work, retirement and pension, income, consumption, assets, and basic community information. CHARLS’ access response rate and data quality are among the top of similar projects in the world, and the data have been widely used and recognized in academia. These empirical data come from the latest public data of CHARLS (2018 national tracking survey data). All the research operations were performed using Stata 16 software.

### 2.2. Theoretical Framework and Variable Selection

Theoretical models of health cost or medical expenditure have developed rapidly in the past two decades [37]. One of them is the Behavioral Model of Health Services Use (BMHSU) proposed by Andersen (1995) [38], which is composed of three factors (including predisposing characteristics, enabling resources, and need) and their sub-variables. It is mainly used to explain individual medical service utilization behavior and its influencing factors and provides theoretical support framework for related research on health service utilization. In this paper, relevant variables are selected from the CHARLS database according to the Andersen model, as shown in Table 1.

Some of the variables are described as follows:

Explanatory variables. As for the utilization of medical services, most scholars tend to evaluate the utilization frequency of medical services and the consumption expenditure of medical services. In terms of medical service consumption expenditure, scholars generally select outpatient expenditure and inpatient expenditure within a certain period of time to refine the total medical expenditure. Outpatient expenditure refers to the medical treatment outpatient expenditure in the past year; inpatient expenditure is the total hospitalization expenditure for the most recent visit within the past year; total medical expenditure is the sum of the most recent outpatient expenditure and inpatient expenditure in the past year. It is important to note here that the reason we drew the distinction between outpatient expenditure and inpatient expenditure is because outpatient expenditure was basically paid by individuals; inpatient expenditure, on the other hand, is largely covered by medical insurance in China, so it makes sense to study them separately.

Physical activity intensity. Does the individual do this type of activity consistently for at least 10 min per week? Inactive is “0,” low-intensity physical activity is “1,” moderate-intensity physical activity is “2,” and high-intensity physical activity is “3.” In the CHARLS database survey, the original question was “Do you usually take this type of activity for at least 10 min every week?”, with type of physical activity including: (1) Vigorous-intensity activity (Vigorous activities can cause shortness of breath. Examples of vigorous-intensity activities include carrying heavy objects, digging, hoeing, aerobic workout, bicycling at a fast speed, riding a cargo bike/motorcycle, etc.); (2) Moderate activity (Moderate activities can make you breathe faster than usual. Examples of moderate activities include carrying light objects, bicycling at a normal speed, mopping, Tai-Chi, and speed walking; (3) Mild activities such as walking (walking from one place to another place at a workplace or home and taking a walk for leisure, sports, exercise, or entertainment). In this paper, the type of physical activity is renamed low-intensity physical activity, moderate-intensity physical activity, and high-intensity physical activity.

Education level. Education level is divided into four categories according to the respondents’ highest level of education (excluding adult education): “0” is no formal education; “1” refers to those who have received primary education or below (including private schools, kindergartens, pre-schools, and primary schools); “2” is education above primary school: secondary school or below; “3” is a college education or above.

Access to medical resources. The number of kilometers traveled from the respondent’s home to a medical facility receiving outpatient or inpatient medical services was reflected in the number of kilometers.

Self-rated health status. Self-rated health status comes from the subjective evaluation of the health status of the respondents in the CHARLS database, and there are five measures for the respondents to evaluate themselves: very good, good, fair, poor, and very poor. In this data processing, the self-rated health status is assigned a 0 for very poor and poor, 1 for fair, and 2 for good and very good.

### 2.3. Empirical Model

According to the preliminary observation of variable data, we find that there are a lot of “0” values in medical expenditure data. In order to avoid the possible endogenous problems caused by this result, this paper adopts a two-part model as an empirical tool.

Before the introduction of health economics analysis, the two-part model was widely used in meteorology and other research fields [39,40,41]). Newhouse and Phelps (1976) [42] provided the first known example of a two-part model in health economics, while afterwards, Duan (1983) [43] used a two-part model to model medical expenditures; it has been widely used in health economics and health service research [44].

The two-part model is mainly used to deal with endogeneity problems caused by medical expenditure with a large number of “0” values. The two-part model includes the probability model of medical treatment and the medical expenditure level model, which are independent of each other; that is, the level of individual medical expenditure has nothing to do with whether they choose medical treatment or not. The first part of the two-part model usually uses the probit model to estimate the probability of individuals choosing to use medical and health services. The second part estimates the level of individual medical expenditure for individuals with medical expenditure.

(1) $$P_{i}(y_{i}>0|x_{i})$$

(2) $$E\left(y_{i}|x_{i},y_{i}>0\right)$$

(3) $$E\left[\log\left(y_{i}|P_{i}>0\right)\right]$$

(4) $$E\left(\log y|x\right)=P_{r}\left(y>0|x\right)E\left(\log y|y>0,x\right)$$

The specific process is as follows. First, the probit model is used to estimate the probability of individuals choosing to use medical and health services (Equation (1)). Second, the medical expenditure level model is used to estimate the medical expenditure of individuals with a positive probability of using medical and health services in the first step (Equation (2)). This formula can be used by ordinary least squares (OLS), generalized linear model (GLM), and other estimation methods.

As Deb et al. (2017) [45] states, “while OLS regressions with a log-transformed dependent variable appear similar to GLM models with a log link, the GLM models are easier to interpret on the raw scale and naturally adjust for heteroskedasticity.” However, in our empirical process, the empirical results obtained by using GLM are not ideal, but the empirical results of the log formalized least squares (LOLS) are better. We will further explore the reasons in future research. At least for now, log formalized least squares (LOLS) has been a good solution to our research problem.

**Figure A1:**
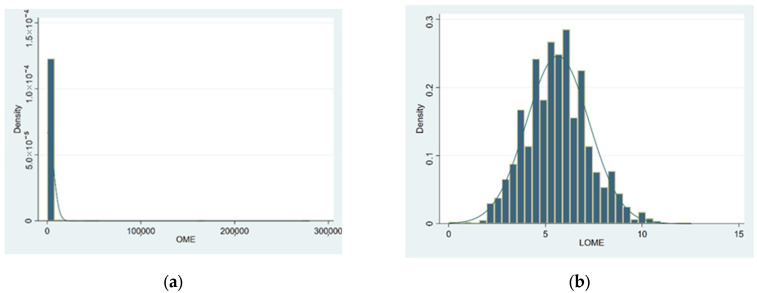
Comparison of normal distribution map of outpatient expenditure before and after logarithmic treatment. (**a**) Normal distribution of outpatient expenditure. (**b**) Normal distribution of outpatient expenditure after logization.

We found that the medical expenditure data itself showed the characteristics of “multi-zero value, large amount gap.” The specific performance is: first, for the vast majority of individuals in a short period of time, the medical expenditure is zero; second, there is a large gap in medical expenditures between individuals with medical expenditures in a short period of time; third, there are very few individuals with very high medical expenditures. For this, we perform logarithmic processing on corresponding variables to correct this skewed distribution, and the effects are as follows (taking outpatient expenditure as an example, Figure A1).

Logarithmic processing can correct the skewed distribution of medical expenditure data. There are also many examples in the existing research results of using the LOLS method to measure medical expenditure in the second part of the two-part model: for example: Lv et al. (2020) used the linear OLS model to estimate the logarithm of non-zero medical expenses in the second part of the expenditure model when studying the relationship between population aging, time near death, and medical expenses based on the data from the follow-up survey of health factors affecting the elderly in China [46], etc. Therefore, the LOLS model is selected for this empirical study.

In detail, according to the characteristics of the applied data, this paper adopts log formalized least squares (LOLS) to correct the skewness distribution of medical expenditure data. Then, the least squares method was used to estimate individual medical expenditure with a positive probability of using medical and health services. Finally, the estimated probability of seeing a doctor in the first step is multiplied by the estimated individual medical expenditure in the second step to obtain the final estimated individual medical expenditure (Equations (3) and (4)).

## 3. Results

### 3.1. Descriptive Statistics of Variables

In this empirical study, a total of 4456 valid samples were selected, with an average age of 62.955 years old. The majority of the samples were female, with education levels generally remaining at the primary school level. The logarithmic per capita annual income of the sample households was 18,917.838, with an average of 1.055 chronic diseases per sample and poor self-rated health status. The average outpatient expenditure of the sample is 748.613 yuan; the average hospitalization expenditure is 6564.428 yuan; and the average total medical expenditure is 7313.041 yuan.

Detailed descriptive statistics of variables are shown in Table 2.

### 3.2. Multicollinearity Test

Before formal empirical analysis, we need to carry out the multicollinearity test of variables (In order to eliminate the interference of multicollinearity on the empirical results, this paper calculates the VIF (variance inflation factor) of all explanatory variables (Table A1)), and the results show that, except for gender variables, the VIF of all the explained variables is around 1.3, far less than 10, so there is no multicollinearity problem between explanatory variables in this study.

In addition, due to the biased distribution of medical expenditure data in our sample data, direct OLS regression often leads to endogenous problems such as selection bias (as shown in Table 3).

By simple OLS regression, we can find that the empirical results are not good. Therefore, this paper uses the two-part model for empirical analysis to deal with the endogeneity problem caused by a large number of “0” value medical expenditures.

### 3.3. Benchmark Empirical Results

The regression results of the two-part model are shown in Table 4.

Outpatient expenditure: Compared to the people with no physical activity, the outpatient expenditure of the people with low physical activity intensity decreased by 24.8% (*p* = 0.053) per week; that of the people with moderate physical activity intensity decreased by 26.4% (*p* = 0.039) per week; and that of the people with high physical activity intensity decreased by 50.7% (*p* = 0.000) per week.

Inpatient expenditure: People with low physical activity intensity had a 0.55% (per week) reduction in inpatient expenditure compared to the people with no physical activity, but the results were not significant. The inpatient expenditure of those with moderate physical activity intensity decreased by 17.4% (*p* = 0.035) per week, and that of those with high physical activity intensity decreased by 28.7% (*p* = 0.001) per week.

Total medical expenditure: Compared to people with no physical activity, the total medical expenditure decreased by 22.4% (*p* = 0.040) per week in those with low physical activity intensity, 40.4% (*p* = 0.000) per week in those with moderate physical activity intensity, and 62.5% (*p* = 0.000) per week in those with high physical activity intensity.

In addition, the results of covariate regression showed that drink could reduce medical expenditure, in which the outpatient expenditure decreased by 11.5% (*p* = 0.044), the inpatient expenditure decreased by 6.8% (*p* = 0.032), and the total medical expenditure decreased by 17.1% (*p* = 0.040). Urban residents have higher medical expenditures, and the results are at the significance level of 1%. As age increases, the probability of outpatient visits decreases; the probability of inpatient visits increases; and the total medical expenditure increases. Men spend more on medical expenditure than women. The distance between hospitals and medical expenditure are proportional, but the influence coefficient is small. For each additional chronic disease, outpatient expenditure increased by 11.9%; inpatient expenditure increased by 4.1%; and total medical expenditure increased by 14.2%, all of which were significant at the 1% significance level.

### 3.4. Robustness Test

**Figure 1:**
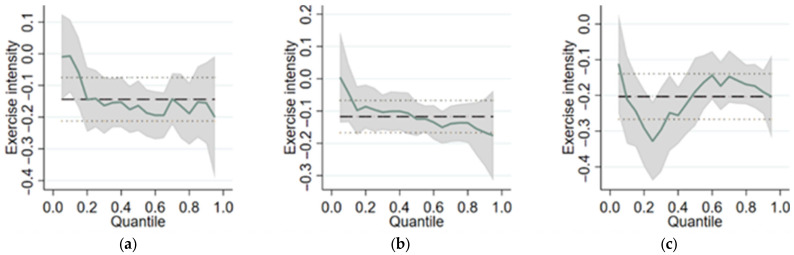
Impact effect of physical activity intensity on medical expenditure based on quantile regression. (**a**) Outpatient expenditure. (**b**) Inpatient expenditure. (**c**) Total medical expenditure. Notes: Dotted lines represent the OLS estimate. The solid line represents the quantile estimate. The shaded area gives the adjusted credible intervals as the parameters were estimated.

After the empirical study, we need to check the robustness of our empirical results by changing variables or empirical methods. We used quantile regression here to test for robustness. The result is shown in Figure 1.

Figure 1 shows the change in the quantile regression coefficient with different quantiles, reflecting the impact effect of physical activity intensity on the distribution of outpatient expenditure, inpatient expenditure, and total medical expenditure in different quantiles. The gray-shaded part in Figure 1 represents a 95% confidence interval. The 95% confidence interval at both ends of the conditional distribution is wider, indicating that the error of the coefficient estimate becomes larger at this time. The thick, dashed, black lines represent the OLS regression estimation results, and the thin, dashed lines at the upper and lower ends represent the confidence interval of OLS regression.

Some information can be found in Figure 1. 

(1) Outpatient expenditure (Figure 1a): the quantile regression coefficient of exercise intensity decreased between 0 and 0.2 and then fluctuated around the level of −0.15. 

(2) Inpatient expenditure (Figure 1b): the quantile regression coefficient fluctuates and decreases from the 0 quantile, but the coefficient is always less than 0.

(3) Total medical expenditure (Figure 1c): the quantile regression coefficient showed a downward trend from the 0.2 quantile, then gradually increased after the 0.2 quantile, and finally tended to −0.17.

(4) In general, in each legend of Figure 1a–c, the quantile regression coefficient is always less than 0. Proper physical activity can reduce the outpatient expenditure. For families with higher inpatient expenditure, there was a greater restraining effect of physical activity on inpatient expenditure. The effect of physical activity on the reduction in the total medical expenditure was highest at the 0.2 quartile, and the effect was discounted later at the 0.2 quartile.

It can be seen from the robustness test that the quantile regression results also recognize the inverse change relationship between physical activity intensity and medical expenditure, so the empirical results are considered to be relatively robust.

## 4. Discussion

In the context of the recent COVID-19 shock and the current worsening economic situation, people are more sensitive to medical expenditure, especially since the high medical expenditure of middle-aged and elderly households increases their risk of falling back into poverty. This concern has also prompted people to pay more attention to physical activity. Therefore, study of the relationship between different physical activity intensities and medical expenditure is of practical significance to the behavioral science of middle-aged and elderly people. This paper selected data from the CHARLS database, classified low, moderate, and high physical activity intensity in middle-aged and elderly people, and discussed their respective effects on medical expenditure.

First of all, the results showed that the medical expenditure of people with high physical activity intensity was significantly lower than that of people with low physical activity intensity in China. This conclusion is similar to that of Marashi et al. (2019) [24] and Watanabe et al. (2018) [26], whose studies were carried out in Japan. It shows that the relationship between physical activity and medical expenditure has reached a consensus among people in some countries.

Secondly, physical activity intensity has a reverse change relationship with the outpatient expenditure, inpatient expenditure, and total medical expenditure. Furthermore, compared to people with no physical activity, the effect of high, moderate, and low physical activity intensity on medical expenditures was increased. In terms of hospital expenditures, for example, the weekly low-intensity exercisers had a 0.55% reduction in hospital expenditures compared to the people with no physical activity, but the results were not significant. Inpatient expenditure was reduced by 17.4% (*p* = 0.035) for moderate-intensity weekly exercisers and 28.7% (*p* = 0.001) for high-intensity weekly exercisers. This conclusion is supported by previous literature such as Araujo et al. (2018) [30] and Wang et al. (2019) [33].

Thirdly, health behaviors such as drinking, smoking, and other living habits are also important factors affecting the medical expenditure of middle-aged and elderly people. Our results showed that drinking can reduce medical expenditures: the outpatient expenditure decreased by 11.5% (*p* = 0.044); inpatient expenditure decreased by 6.8% (*p* = 0.032); and total medical expenditure decreased by 17.1% (*p* = 0.040). This finding is consistent with the research conclusion of Song et al. (2020) [36]. In addition, the outpatient expenditure, inpatient expenditure, and total medical expenditure of urban residents were all higher than rural residents, and the results were at the significance level of 1%. As age increases, the probability of outpatient visits decreases; the probability of inpatient visits increases; and the total medical expenditure increases. The distance to the hospital is directly proportional to the medical expenditure, which is similar to Xu et al. (2022) [47]. In addition, for each additional chronic disease, the outpatient expenditures increased by 11.9%; inpatient expenditures increased by 4.1%; and total medical expenditures increased by 14.2%, all of which were significant at the 1% level of significance.

In conclusion, both the two-part model and the robustness test of quantile regression confirm the inverse relationship between physical activity intensity and medical expenditure in middle-aged and elderly people, which also provides some insights for government policies, especially in the context of the recent end of the COVID-19 pandemic [23]. Governments should increase the motivation of the elderly to exercise more by making them aware of the impact of exercise intensity on their healthcare costs [48]. At the same time, provide more places for the elderly to exercise, planning special exercise areas for the elderly in community playgrounds such as a dancing square to increase their intensity of aerobic exercise.

At the end of the paper, it must be mentioned that our study still has some limitations. For example, in the CHARLS database survey, the original question “Do you usually take this type of activity for at least 10 min every week?”, is the 10 min every week question reasonably designed? While this question is not a problem of our own making and is the responsibility of the CHARLS database research team, we also need to think about the future. In addition, our empirical results in Table 4 show that drinking and smoking reduce health expenditure, which seems counterintuitive. We believe that it may be because individuals who drink or smoke pay less attention to their health than other individuals, or because their family members buy more health insurance due to their health concerns, which leads to them paying less attention to their health and reducing medical expenditures. In order to explore this question more clearly, we will do further research in the next paper.

## Figures and Tables

**Table 1:** Variable selection and assignment.

	Variable	Variable Definition and Assignment
Explained variable	Total medical expenditure	Sum of the expenditure of the latest outpatient + inpatient in the past year.
	Inpatient expenditure	Amount spent on the most recent inpatient within the past year.
	Outpatient expenditure	Amount of the most recent outpatient expenditure within the past year.
Explanatory variables	Physical activity intensity	Does the individual do this type of activity consistently for at least 10 min per week? Inactive is “0”, low-intensity physical activity is “1”, moderate-intensity physical activity is “2”, and high-intensity physical activity is “3”.
	Smoking	Do you smoke? “0” has never smoked, “1” has smoked but quit, “2” is smoking.
	Drinking	The frequency of drinking in the past year. “0” does not drink, “1” does not drink more than once a month, “2” more than once a month.
Predisposing characteristics	Residential address type	Township central area/village and special area is “0”, and the main urban area/urban junction/town center/township junction is “1”.
	Age	Persons older than 45 years of age with actual birth date ending in 2018. Individuals under the age of 45 were excluded.
	Gender	“0” for females and “1” for males.
	Education Level	“0” is no formal education; “1” refers to those who have received primary education or below (including private schools, kindergartens, pre-schools and primary schools); “2” is the education above primary school, secondary school or below; “3” is a college education or above.
	Marital status	Unmarried (never married, cohabitation), divorced (separated as a spouse, divorced, widowed) is “0”, married is “1”.
Enabling resources	Access to medical resources	Distance from a respondent’s home to a medical institution that receives outpatient or inpatient care.
	Per capita annual household income	The ratio of the total wage income and transfer payments received by all members of the household, the sum of household agricultural income, income from self-employment or private enterprise, and the sum of household public transfer payments to the size of the household during the past year.
	Social medical insurance participation status	“1” represents participating in urban and rural residents’ medical insurance (including the New Rural Cooperative Medical Scheme), “2” represents participating in urban employees’ medical insurance, and 0 “represents not participating in the above medical insurance.
Need	Number of chronic diseases	Individuals with hypertension, diabetes, cancer and other malignancies, chronic lung diseases, arthritis or rheumatism, CVD diseases, and other chronic diseases.
	Self-rated health	The self-rated health status was given as “0”, “Very poor” and “poor”, “1” for “fair”, and “2” for “good” and “Very good”.

**Table 2:** Results of descriptive statistics of variables.

Variable	Variable Meaning	Obs (Observation)	Mean	Std. Dev. (Standard Deviation)
OME	Outpatient expenditure	4456	748.613	5638.465
IME	Inpatient expenditure	4456	6564.428	26,758.342
ME	Total medical expenditure	4456	7313.041	27,518.384
exercise	Physical activity intensity	4456	1.781	0.970
	0 No activity	439 (9.85%)		
	1 Light activity	1374 (30.83%)		
	2 Moderate activity	1369 (30.72%)		
	3 Intensive activity	1274 (28.59%)		
smoke	Smoking	4456	0.609	0.812
	0 No cigars	2679 (60.12%)		
	1 Quit	839 (18.83%)		
	2 Still	938 (21.05%)		
drink	Drinking	4456	0.489	0.815
	0 No drink	3201 (71.84%)		
	1 Drink but less	333 (7.47%)		
	2 Drink more than once a month	922 (20.69%)		
address	Residential address type	4456	0.295	0.456
	0 Village	3412 (70.51%)		
	1 City/Town	1314 (29.49%)		
age	Age	4456	62.955	9.931
gender	Gender	4456	0.452	0.498
	0 Female	2443 (54.82%)		
	1 Male	2013 (45.18%)		
education	Education Level	4456	1.138	0.772
	0 No formal education	967 (21.70%)		
	1 Elementary school	2004 (44.97%)		
	2 Middle school	1390 (31.19%)		
	3 College and above	95 (2.13%)		
marital	Marital status	4456	0.848	0.359
	0 Unmarried	679 (15.24%)		
	1 Married	3777 (84.76%)		
distance	Access to medical resources	4456	31.590	144.176
lnmhi	Per capita annual household income	4456	8.998	1.506
ill	Number of chronic diseases	4456	1.055	1.223
selfh	Self-rated health	4456	0.683	0.672
	0 Poor	1935 (43.42%)		
	1 Fair	1997 (44.82%)		
	2 Good	524 (11.76%)		
insurance	Medical insurance	4456	1.049	0.481
	0	413 (9.27%)		
	1	3413 (76.59%)		
	2	630 (14.14%)		

**Table 3:** OLS regression test.

		Outpatient Expenditure	Inpatient Expenditure	Total Medical Expenditure
Level value	Coefficient values	−161.67 * (−1.73)	−1340.263 *** (−3.06)	−1501.94 *** (−3.34)
	Ramsey inspection	5.82 ***	57.82 ***	48.75 ***
Logarithmic transformation	Coefficient values	−0.14 *** (−4.10)	−0.12 *** (−4.59)	−0.20 *** (−6.25)
	Ramsey inspection	57.39 ***	15.93 ***	48.89 ***
Square root transformation	Coefficient values	−0.49 (−1.24)	−5.57 *** (−5.49)	−6.06 *** (−6.24)
	Ramsey inspection	0.12	35.00 ***	46.39 ***

Note: Numbers in parentheses are *t*-values. *** *p* < 0.01, * *p* < 0.1.

**Table 4:** Results of the two-part model.

Variables	Outpatient Expenditure		Inpatient Expenditure		Total Medical Expenditure	
	Probit	regress_log	Probit	regress_log	Probit	regress_log
1. Low physical activity intensity	0.075	−0.248 *	−0.153 **	0.005	−0.138	−0.224 **
	(0.071)	(0.128)	(0.076)	(0.079)	(0.234)	(0.109)
2. Moderate physical activity intensity	0.167 **	−0.264 **	−0.230 ***	−0.174 **	−0.354	−0.404 ***
	(0.073)	(0.128)	(0.076)	(0.082)	(0.233)	(0.111)
3. High physical activity intensity	0.208 ***	−0.507 ***	−0.257 ***	−0.287 ***	−0.230	−0.625 ***
	(0.074)	(0.129)	(0.078)	(0.086)	(0.242)	(0.114)
smoke	−0.028	−0.064	−0.048	−0.082 **	−0.024	−0.126 **
	(0.032)	(0.055)	(0.033)	(0.040)	(0.089)	(0.050)
drink	0.041	−0.115 ***	−0.087 ***	−0.068 **	−0.003	−0.171 ***
	(0.026)	(0.044)	(0.027)	(0.032)	(0.074)	(0.040)
address	−0.081 *	0.245 ***	0.091 *	0.270 ***	0.086	0.373 ***
	(0.048)	(0.080)	(0.049)	(0.059)	(0.142)	(0.073)
age	−0.022 ***	0.001	0.024 ***	−0.003	−0.014 **	0.024 ***
	(0.002)	(0.004)	(0.002)	(0.003)	(0.006)	(0.003)
gender	−0.185 ***	0.329 ***	0.310 ***	0.232 ***	−0.076	0.608 ***
	(0.057)	(0.096)	(0.058)	(0.069)	(0.162)	(0.088)
education	0.051 *	0.065	−0.025	0.058 *	0.076	0.017
	(0.029)	(0.048)	(0.030)	(0.035)	(0.085)	(0.045)
marital	−0.068	0.220 **	0.057	0.071	0.157	0.180 **
	(0.057)	(0.096)	(0.059)	(0.067)	(0.143)	(0.088)
distance	−0.002 ***	0.004 ***	0.002 ***	0.001 ***	0.006 *	0.002 ***
	(0.000)	(0.000)	(0.000)	(0.000)	(0.003)	(0.000)
lnmhi	0.019	0.032	−0.025 *	0.056 ***	0.006	0.020
	(0.014)	(0.023)	(0.014)	(0.017)	(0.041)	(0.021)
ill	−0.019	0.119 ***	0.078 ***	0.041 **	0.082	0.142 ***
	(0.016)	(0.026)	(0.017)	(0.019)	(0.054)	(0.025)
selfh	−0.022	−0.211 ***	−0.144 ***	−0.080 **	−0.093	−0.307 ***
	(0.030)	(0.050)	(0.030)	(0.037)	(0.083)	(0.046)
insurance	−0.028	0.154 **	0.113 ***	0.144 ***	0.166	0.251 ***
	(0.043)	(0.070)	(0.043)	(0.052)	(0.121)	(0.065)
Constant	1.454 ***	4.987 ***	−1.138 ***	7.909 ***	2.877 ***	5.283 ***
	(0.217)	(0.353)	(0.220)	(0.263)	(0.630)	(0.330)
Observations	4456	4456	4456	4456	4456	4456

Note: The number in the first row of each variable is the regression coefficient, and the number in parentheses is the standard error. *** *p* < 0.01, ** *p* < 0.05, * *p* < 0.1.

## Data Availability

Please refer to: http://charls.pku.edu.cn/index.html (accessed on 25 July 2021). More data that if the reader has a personal request, we will provide it to him.

## Appendix B

**Table A1:** Results of multicollinearity test.

Variable	VIF (Variance Inflation Factor)	1/VIF
gender	2.160	0.462
smoke	1.900	0.526
education	1.360	0.737
age	1.330	0.755
address	1.270	0.786
drink	1.250	0.801
lnmhi	1.200	0.832
exercise	1.150	0.869
marital	1.140	0.874
insurance	1.000	0.996
selfh	1.130	0.881
ill	1.110	0.905
distance	1.060	0.947
Mean VIF	1.310	
